# Drug Resistance Mutations (DRMs) for Long-Acting Injectable Cabotegravir and Rilpivirine (CAB/RPV LAI) in the HIV-1 Subtype A6 Epidemic in Poland

**DOI:** 10.3390/microorganisms13020321

**Published:** 2025-02-01

**Authors:** Andrzej Załęski, Agnieszka Lembas, Tomasz Dyda, Joanna Osińska, Joanna Jabłońska, Justyna Stempkowska-Rejek, Justyna Orzechowska, Alicja Wiercińska-Drapało

**Affiliations:** 1 Hospital for Infectious Diseases in Warsaw, Wolska 37 Street, 01-201 Warsaw, Poland; andrzejzaleski84@wp.pl (A.Z.);; 2 Department of Infectious Diseases, Tropical Diseases and Hepatology, Medical University of Warsaw, Żwirki i Wigury 61 Street, 02-091 Warsaw, Poland; 3 Molecular Diagnostics Laboratory, Hospital for Infectious Diseases in Warsaw, Wolska 37 Street, 01-201 Warsaw, Poland; 4 Infectious Diseases Clinical Ward in Ostróda, Department of Family Medicine and Infectious Diseases, University of Warmia and Mazury in Olsztyn, Oczapowskiego 2 Street, 10-719 Olsztyn, Poland; 5 Department of Infectious Diseases and Hepatology, Medical University of Lublin, Aleje Racławickie 1 Street, 20-059 Lublin, Poland; 6 Clinical Department of Infectious Diseases, Medical Center in Łańcut, College of Medical Sciences, University of Rzeszów, Rejtana 16c Street, 35-310 Rzeszów, Poland

**Keywords:** HIV subtype, A6, DRMs, drug resistance, ART, LAI, CAB/RPV

## Abstract

HIV subtype A6 with the L74I polymorphism, which increases the risk of cabotegravir/rilpivirine treatment failure, causes more and more infections in Poland. In this multicenter, observational, cross-sectional study (2023–2024), we analyzed viral subtypes and drug-resistance mutations to drugs used for long-acting injectable antiretroviral treatment and pre-exposure prophylaxis. Among 357 people with HIV, 247 (69%) were Polish nationals, and 102 (29%) were from former Soviet Union countries. Of the 357 people included, 159 (45%) had subtype B, and 177 (50%) had subtype A6 infections, with 165 (87%) of the latter characterized by the L74I polymorphism. Subtype A6 was more frequent in women (66% vs. 46% in men, *p* < 0.05) and among people from former Soviet countries (77% vs. 39% in Polish nationals, *p* < 0.05). About 40% of people had either drug-resistance mutations for cabotegravir/rilpivirine or HIV A6 subtype with the L74I polymorphism; 4.5% had both of these conditions. Compared to subtype B infections, subtype A6 infections were characterized by more frequent major transmitted drug-resistance mutations for non-nucleoside reverse transcriptase inhibitors (8.5% vs. 1.9%, *p* = 0.007) and rilpivirine (5.1% vs. 0.6%, *p* = 0.016). Due to the frequent occurrence of the L74I polymorphism and drug-resistance mutations in HIV A6 subtype infection, about 40% of people with HIV in Poland may be at risk of long-acting injectable treatment failure.

## 1. Introduction

The first cases of human immunodeficiency virus (HIV) infection were reported in 1981 in Los Angeles, USA. HIV was initially identified in 1983, and its etiological connection with acquired immunodeficiency syndrome (AIDS) was discovered in 1984 [1]. Since then, around 42 million people have died of AIDS, and around 40 million are currently infected with HIV worldwide [2]. At the beginning of the global HIV pandemic, antiretroviral therapy (ART) was unavailable, with most deaths among HIV-positive people caused by AIDS-related diseases. Currently, the life expectancy of people with HIV (PWH) receiving effective ART is similar to the general population [3]. Treatment options, among others, include nucleoside reverse transcriptase inhibitors (NRTIs), nonnucleoside reverse transcriptase inhibitors (NNRTIs), protease inhibitors (PIs) and integrase strain transfer inhibitors (InSTIs). The use of InSTIs and other drugs with high genetic barriers simplified treatment schemes decreased the risk of treatment failure and enabled two-drug combinations, including long-acting injection formulations [4]. Currently, the LAI combination of cabotegravir (CAB) and rilpivirine (RPV) is recommended as a switch therapy in virologically suppressed patients. Additionally, CAB injections serve as preexposure prophylaxis [4,5,6].

The differential genetic characteristics of HIV subtypes may influence HIV transmission, treatment outcomes, and disease progression [7]. Among the two HIV types, HIV-1 accounts for about 95% of all infections worldwide. HIV-1 is classified into 4 groups (M, N, O, and P), of which group M is the most widespread. Group M is divided into nine distinct subtypes: A, B, C, D, F, G, H, J, and K. Subtype C accounts for almost half of HIV-1 infections worldwide, whereas subtypes B and A for 12% and 10%, respectively. Subtypes can combine to form a hybrid: circulating recombinant form (CRF) or unique recombinant form (URF). CRF02_AG and CRF01_AE, count for 13% of infections [8]. Subtype A remains the most prevalent strain (>50%) in Russia, former Soviet Union countries (FSU), and parts of East Africa, whereas subtype B predominates in Western Europe (75%), the Americas, and Oceania. Subtype C is detected primarily in Southern Africa and India; CRF01_AE, in Asia; and CRF02_AG, in Western Africa. Recent studies based on near-full-length genome sequencing highlight the growing importance of recombinant variants and subtype C [9,10]. Subtype A, of low diversity, and a new circulating recombinant form derived from it, CRF03_AB and CRF02_AG, have emerged in the FSU [11].

Different HIV-1 subtypes predominate in certain geographic areas, but their distributions are becoming increasingly heterogeneous as the pandemic progresses [8,12]. Over the past decades, an increasing prevalence of non-B subtypes in Europe has been reported, and non-B subtypes have become endemic in the European population, not only in migrants from FSU. In Poland, the increasing number of subtype A HIV-1 infections, which now constitute the second most prevalent circulating subtype (up to 10%), has been associated with migration from the conflict in Ukraine, where subtype A is most prevalent [13,14]. Data from recent studies have shown that HIV-1 subtype A6 with the L74I polymorphism is a risk factor for treatment failure with long-acting injectable CAB/RPV [15]. In addition, treatment failure can be caused by drug-resistance mutations (DRMs), particularly in people exposed to few active agents [16]. Therefore, increasing frequencies of treatment-resistant subtypes and DRMs may lower the efficacy of long-acting injectable CAB/RPV and preexposure prophylaxis in certain populations, including Poland and FSU. In this study, we analyzed the frequency of treatment-resistant subtypes and DRMs associated with treatment failure with a focus on long-acting therapy. This work is part of active surveillance, which is crucial for understanding the factors involved in the transmission of HIV resistance and for designing effective local treatment guidelines.

## 2. Materials and Methods

### 2.1. Study Design

We conducted a multicenter, observational, cross-sectional study. Patient data were extracted from a 2-year period spanning from 2023 to 2024. The recruitment sites were four clinical centers in Poland (Warsaw, Ostróda, Lublin, and Rzeszów). There were no differences in the profiles of the clinical centers (each consists of an outpatient clinic and hospital department). The inclusion criteria were HIV-1 infection and aged 18 years or older.

### 2.2. Clinical Assesment

We collected data such as age, sex, nationality, route of transmission (heterosexual route, through homosexual and bisexual contacts (men having sex with other men—MSM), through intravenous drug use (IDU) or vertical route) and clinical findings (AIDS stage, recent HIV infection). According to the latest European AIDS Clinical Society (EACS) Guidelines for the management of people living with HIV in Europe and European Centre for Disease Prevention and Control (ECDC), AIDS was defined as the presence of any illness from AIDS-defining condition list provided by Centers for Disease Control and Prevention (CDC) and recent HIV infection was defined as diagnosed up to 6 months after infection: on the basis of seroconversion or diagnosis of acute HIV infection or individuals’ medical history [4,17,18]. In all patients included in the study, the presence of HIV-1 DRMs to all antiretrovirals, particularly to NNRTIs and InSTIs, was evaluated. The drug resistance was performed in accordance with the Stanford University HIV Drug Resistance Database [19].

### 2.3. Molecular Investigation

HIV-1 drug resistance genotyping of the HIV-1 pol region, including protease (PR), reverse transcriptase (RT), and integrase (INT) coding genes, was performed using the DeepChek HIV-1 PR/RT/INT Drug Resistance Assay (ABL-Advanced Biological Laboratories Diagnostics S.A., Luxembourg), following the manufacturer’s instructions. Template HIV-1 RNA was manually extracted from plasma samples collected between 2023 and 2024 from HIV-1-positive patients with viral loads exceeding 1000 copies/mL (the test cutoff). The HIV-1 RNA purification was conducted using the QIAamp Viral RNA Mini Kit (Qiagen, Hilden, Germany). Amplification of HIV-1 pol region fragments was achieved via reverse transcription preceding polymerase chain reaction (RT-PCR) followed by a nested PCR step to enhance sensitivity and specificity. The targeted regions included protease gene (codons 1–99), codons 1–320 of the reverse transcriptase (RT) gene, and codons 20–280 of the integrase (INT) gene. The obtained amplicons quality and purity were assessed by visual inspection of the expected molecular sizes of nested PCR products as follows: 937 bp for reverse transcriptase, 520 bp for protease, 670 bp for integrase observed as result of gel electrophoresis. Nested PCR products were enzymatically purified using an Exo–SAP mix (Exonuclease I and Shrimp Alkaline Phosphatase). The purified products were diluted as necessary, fluorescently labeled with appropriate terminators, and sequenced using an ABI Prism 3500 Genetic Analyzer (Applied Biosystems, Waltham, MA, USA) and the Data Collection Software v4. The sequencing raw data were uploaded to the ViroScore v.3 DeepChek-HIV IVD software (ABL Diagnostics S.A., Luxembourg). Quality control was performed to ensure a required quality threshold of sequence chromatograms were assembled. The software was used to analyze the obtained HIV-1 sequence bases on an updated database of HIV drug resistance-associated mutations and their latest interpretations rules. The assay was validated for HIV-1 subtype B but is also capable of amplifying other subtypes and recombinant forms, such as CRF02_AG. Drug resistance-associated mutations were identified for each sample. Quality-checked pol sequences were also submitted to the Stanford University HIV Drug Resistance Database (http://hivdb.stanford.edu accessed on 20 January 2024) for comparative analysis and interpretation of the genetic variants detected. Subtyping, identification of circular recombinant forms (CRFs), and drug resistance mutation analysis were further enhanced using online tools provided by the Stanford University HIV Drug Resistance Database and the REGA HIV-1&2 Subtyping Tool. Nucleotide alignments were performed using the MEGA v.12 software package, followed by manual editing. The Hasegawa–Kishino–Yano model with discrete gamma distribution (HKY+G) was determined to be the best-fitting substitution model for the phylogenetic alignments. Phylogenetic robustness was tested using the online PhyML algorithm, applying nonparametric bootstrap resampling with 100 replicates. HIV-1 viral load in plasma samples was quantified using the Cobas 5800 HIV-1 Test (Roche, Basel, Switzerland). This assay targets two distinct regions of the HIV-1 genome (gag and LTR). The analytical performance parameters were as follows: sensitivity: 13.2 copies/mL, linear range: 20 copies/mL to 1.0 × 10^7^ copies/mL, specificity: 100%.

### 2.4. Statistical Evaluation

The statistical evaluation of the collected data was performed in subgroups of PWH with the presence of drug class-specific major and accessory DRMs and polymorphisms (including L74I) associated with ART resistance in A6 and B subtypes. The analysis was also performed in the subgroups of DRMs to drugs used in LAI ART (CAB and RPV). The Shapiro–Wilk test was performed to verify the normality of the distributions of the analyzed variables. Student’s *t* test or the Mann–Whitney U test was used to evaluate the difference in the mean values of continuous variables, and the χ^2^ test or Fisher’s exact test was performed for categorical variables. A p value of <0.05 was considered to indicate statistical significance. Statistical analyses were performed using Python 3.7 software and the Statistica 13.1 program (StatSoft Poland, Kraków, Poland).

### 2.5. Bioethics

This study was conducted in accordance with the guidelines of the Declaration of Helsinki, was approved by the Bioethics Committee at the Medical University of Warsaw (AKBE/72/2023) on 6 February 2023, and it constitutes part of a study registered on ClinicalTrials.gov (NCT06661317). Informed consent was obtained from all the subjects involved in the study. All patients data analyzed were fully anonymized.

## 3. Results

### 3.1. Cohort Characteristics

**Figure 1:**
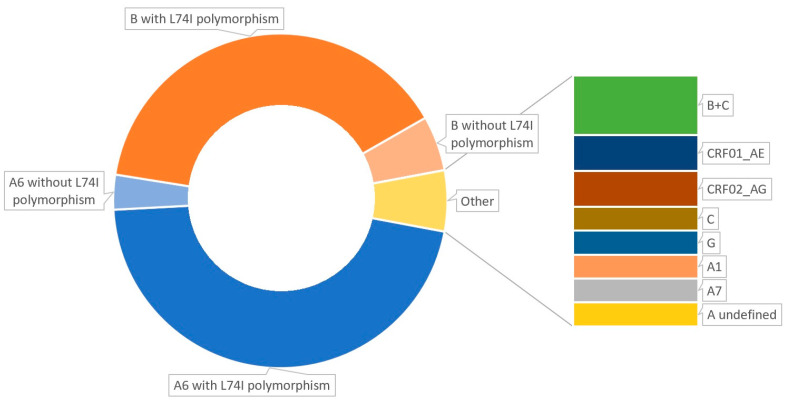
HIV subtype and L74I polymorphism distributions in the study group.

**Figure 2:**
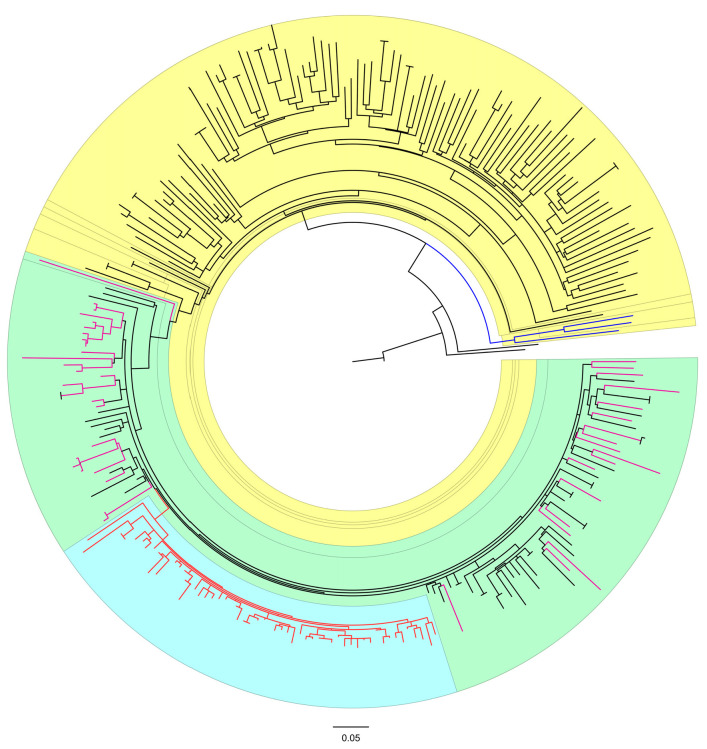
Maximum likelihood phylogenetic analysis of Polish HIV-1 subtype A pol sequences. Inferred phylogenetic relationships. The sequences of HIV-1 subtype A1 from Los Alamos database are presented in black. The HIV-1 subtype A1 sequences from isolates with African origin have been highlighted in yellow, A6 subtype have been highlighted in light green. Singular introduction of A6 were marked purple are dispersed as monophyletic clades. The cluster, which is probably a result of the former, singular introduction of HIV-1 A6, was highlighted in light blue and marked in red. Three sequences of HIV-1 A2 subtype isolates obtained from the Los Alamos database marked in dark blue.

We analyzed data from 357 PWH, among whom 177 patients had HIV subtype A6 (49.58%), 159 individuals (44.54%) had subtype B, and 5 had subtype B+C; 2 patients each had A1, A7, undefined subtype A, C, or G (n = 10), and 3 patients each had CRF01_AE or CRF02_AG (n = 6). 54.24% of all A6 were diagnosed in Polish PWH. Subtype A6 was more frequent in women (66.10% vs. 46.31% in men, *p* < 0.05) and among people from former Soviet countries (77.45% vs. 38.87% in Polish nationals, *p* < 0.05). The baseline characteristics of the study population are presented in Table 1 and Figure 1 and Figure 2.

Data concerning the L74I polymorphism were available for 322 out of all 357 patients (90.20%). Among PWH with the B subtype, L74I was present in 28.57% (40/140), whereas the A6 subtype was present in 86.67% (165/177) and was more common (*p* < 0.001). There were no statistically significant differences between the sizes of these two cohorts (*p* = 0.102).

### 3.2. Presence of DRMs

The presence of major and accessory HIV DRMs was analyzed. The results are presented in Table 2 and Figure 2.

### 3.3. Presence of DRMs to LAI CAB+RPV and CAB PrEP

**Figure 3:**
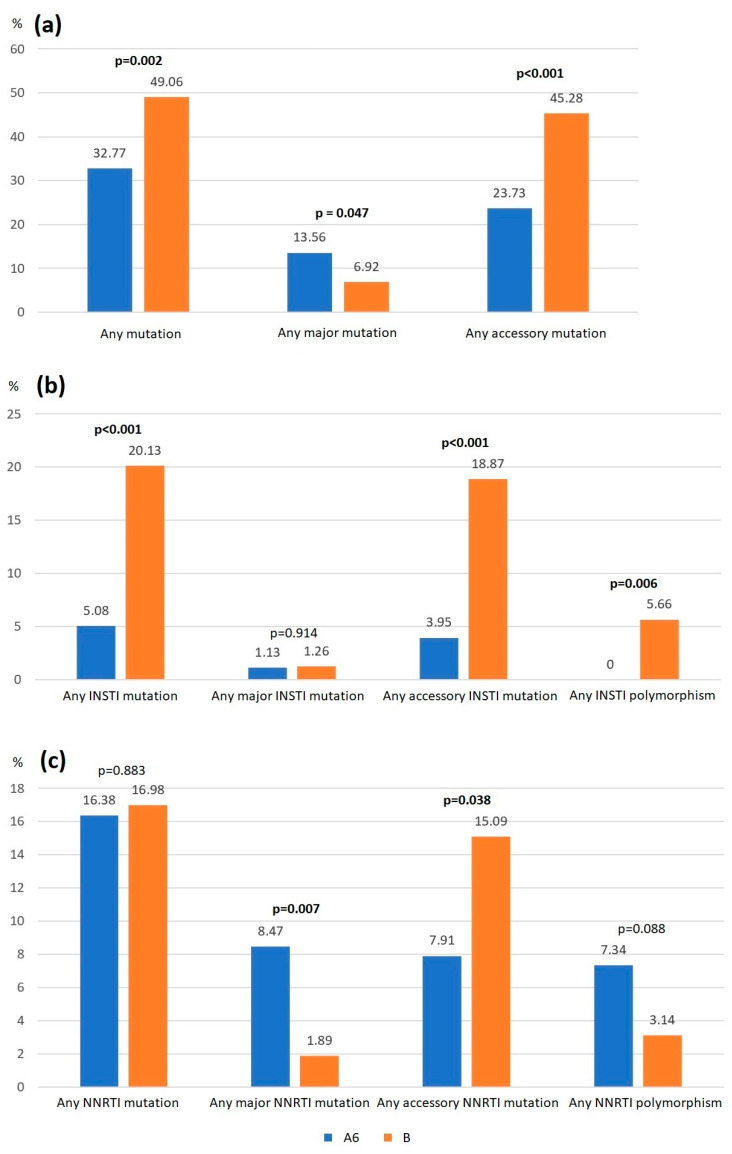
The percentage of HIV drug resistance mutations according to HIV subtype. (**a**) The percentage of all HIV drug resistance mutations depending on the HIV subtype. (**b**) The percentage of HIV drug resistance mutations to InSTIs depending on the HIV subtype. (**c**) The percentage of HIV drug resistance mutations to NNRTI depending on the HIV subtype.

The presence of HIV DRMs to CAB and RPV was analyzed. The major CAB mutations were E138K and R263K (most frequent—0.56% each), N155H and G118R, and the accessories were L74F/M (most frequent—0.84%), S153Y, G149A, and S230R. We identified major RPV mutations, namely V179T/E (most frequent—2.52%), E138K, Y181C, G190S and K101E. The identified accessory RPV mutations were V106I (most frequent—5.32%), V179D, E138A, and V90I. The collective results are presented in Table 3 and Figure 3.

In the analyzed population, there were no patients with HIV DRMs to both CAB and RPV. There were 55 (15.40%) patients with HIV DRMs to either CAB or RPV, including 16 major mutations and 39 accessory ones.

**Figure 4:**
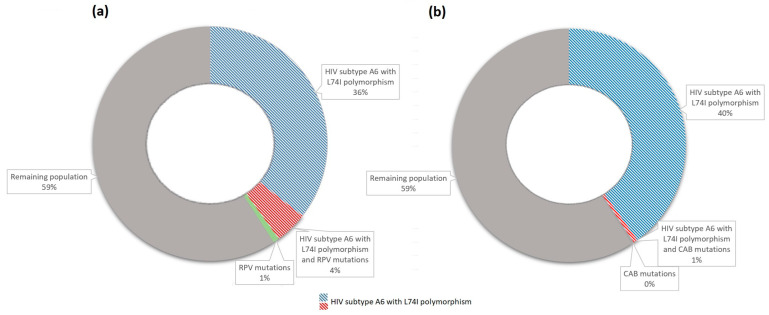
Distribution of HIV drug resistance mutations to LAI ART in the study group. (**a**) The distribution of HIV drug resistance mutations to RPV in the study group. (**b**) The distribution of HIV drug resistance mutations to CAB in the study group.

There were 40.89% (146/357) of patients with either RPV DRMs or the HIV A6 subtype with the L74I polymorphism (143 patients with HIV subtype A6 with the L74I polymorphism and 3 patients with RPV mutations: 1 major, 2 accessory) and 4.48% (16/357) of patients with both HIV subtype A6 with the L74I polymorphism and RPV DRMs (8 major, 8 accessory). There were 40.34% (144/357) patients with either CAB DRMs (L74F excluded) or the HIV A6 subtype with the L74I polymorphism (143 patients with HIV subtype A6 with the L74I polymorphism and 1 patient with 1 major CAB mutation) and 0.56% (2/357) patients with both HIV subtype A6 with the L74I polymorphism and 2 major CAB DRMs (Figure 4).

## 4. Discussion

### 4.1. Changing HIV Subtype Epidemiology in Poland

Although accurate data are scarce, in recent years, Eastern Europe and Central Asia have shown an alarming increase in annual HIV infection rates; as of mid-2017, Russia, with more than 1.16 million estimated infections, is among the countries accounting for almost 90% of all new HIV infections worldwide [6,20,21]. The collapse of the Soviet Union in the early 1990s led to massive transmigration within the FSU, which, in turn, led to the spread of HIV infections, among others. Newly diagnosed HIV-1 infections in Ukraine before the Russian invasion in 2022 numbered approximately 16,000 cases every year. The estimated incidence rate of HIV in FSU is 32.6 per 100,000 people. These data stand in opposition to HIV incidence rates in Western Europe, which range from 2.3 to 3.7 per 100,000 inhabitants [22].

While certain HIV subtypes predominate by geographic area, their distribution changes as the pandemic progresses. In recent decades, in the geographical context of Europe and Russia, subtype A has remained the most prevalent HIV-1 subtype in FSU, whereas subtype B has predominated in Western and Central European countries, such as Poland (75%) [8,11,13,23]. Owing to continuous local and global changes, the idea that subtype B is the primary relevant subtype in Western Europe and North America no longer reflects the current epidemiological situation. Recently, an increasing prevalence of non-B subtypes has been reported in Europe, becoming endemic not only among migrants from FSU countries but also among non-foreign-born European citizens [13,23,24]. The FSU HIV-1 A6 subtype, which evolved from subtype A1 of African origin and was first identified among IDUs in Russia, is responsible for rapidly expanding epidemics not only in FSU but also outside the FSU region. For this reason, many European countries, including Poland and Germany, have experienced an increase in the frequency of HIV-1 A6 subtype infections in recent years [13,14,25].

High-risk behaviors associated with HIV A6 transmission are reported mainly for FSU sequences from Russia and Ukraine and include heterosexual contact, IDU, and sexual contact among MSM [23,26]. In contrast to Western Europe, in Ukraine, before the Russian invasion, almost 65% of annual HIV-1 transmissions occurred through heterosexual contact, 30% among IDUs, and only 3% among MSM [14,22]. Past migration from Ukraine has begun the spread of the A6 subtype. Recent Polish studies demonstrated that almost 70% of HIV-1 A6 clades in Poland originated from Ukraine, followed by 30% from Russia, with introductions spanning from 1993 to 2008 and enhanced by war migration. Currently, in Poland, the A6 subtype accounts for 8.66% of all HIV infections and is present in the majority (89%) of infected Ukrainian migrants, among whom over one-third are women; moreover, transmission among MSM is the dominant route of infection (73%) [13,27,28,29].

The current spread of the A6 subtype in Poland is irrefutable. The fact that almost half (49.58%) of all HIV infections in our cohort were caused by the A6 subtype, in comparison to 8.66% in 2015–2019, lends support to the statement [13]. As reported previously, subtype A6 was present more frequently (*p* < 0.05) in individuals from FSU (77.45% vs. 38.87% in Polish nationals, *p* < 0.05) and women (66.10% vs. 46.31% in men, *p* < 0.05), which confirms the common heterosexual route of transmission (32.77% for subtype A). Interestingly, Polish patients (82.39%), mainly MSM (74.21%), predominated subtype B cases, which is consistent with previous data [6,8,13,22]. However, more than half (54.24%) of all A6 infections were also diagnosed in individuals of Polish nationality, which, along with an increasing total number of A6 infections, is evidence of the accomplished epidemic spread of the A6 subtype outside the FSU region. The A6 and B cohorts did not differ significantly regarding the number of individuals, age of participants, other routes of spread (vertical, IDU), or diagnosed stage of HIV infection (AIDS, recent HIV infection), which suggests an unlikely population selection bias in the study. Owing to continuous changes in HIV epidemiology and a growing body of evidence that the epidemic in Poland has started to involve groups that are less conscious of HIV infection risk (heterosexual contacts), up-to-date surveillance is essential for HIV management.

### 4.2. A6 Subtype Genetic Diversity

The genetic barrier, defined as the number of mutations required to overcome drug selective pressure, is an important factor in HIV drug resistance development [30]. Studies have shown that the virological outcomes of ART can be related not only to resistance mutations but also to subtype-specific differences, highlighting the importance of analyzing the HIV subtype features for an improved understanding of viral subtype variability in the occurrence of DRMs, especially in cases of emergence of HIV drug resistance subtypes, such as A6. Generally, differences in the magnitude of resistance depend on genotypic context and are mutation- and subtype-related [7,15,23,24,30,31].

#### 4.2.1. Significance of the L74I Polymorphism in the A6 HIV-1 Subtype

The L74I integrase polymorphism, as a sign of viral genetic variability, occurs in 7% of the integrase genes of HIV sequences in the Los Alamos National Laboratory database [32]. L74I is particularly prevalent in individuals from Russia and FSU, being present in between 93% and 100% of HIV-1 subtype A isolates and being the consensus amino acid of the A6 clade [30,32,33]. It does not appear to have been selected by any InSTI; rather, it was selected at the early stage of the A6 epidemic and spread in Russia as a founder effect [12,30].

HIV-1 subtype A6, containing the L74I polymorphism, is associated with the confirmed virological failure of LAI CAB/RPV. L74I alone does not cause InSTI resistance, and most patients (93%) with the baseline L74I polymorphism can maintain virological suppression during CAB/RPV treatment [15]. However, substitutions at this position, in combination with other integrase mutations, can reduce susceptibility to CAB. Other changes at position 74, such as L74M, also impact CAB resistance. L74M in combination with integrase mutations at positions 140 and 148 reduces CAB susceptibility 53- to 220-fold. Recent research has shown a lower genetic barrier for L74M and L74I mutations in the A6 subtype, suggesting a lower barrier for InSTI resistance [25,30,34]. Moreover, A6, which harbors the L74I polymorphism, confers greater replication capacity to recombinant viruses expressing the HIV-1 A6 integrase when present together with InSTI resistance mutations at defined positions. Hu et al. demonstrated that the presence of L74I conferred increased fitness to recombinant viruses expressing the HIV-1 A6 integrase when present with G118R, G140R, Q148H, and R263K. Thus, the L74I polymorphism may facilitate the emergence of CAB resistance in subtype A6 viruses by increasing the replication capacity of variants carrying InSTI DRMs [35]. Cutrell et al. suggested that both HIV-1 subtype A6/A1 and L74I are required to increase the odds of confirmed viral failure [15]. In the context of drug resistance, RPV DRMs constitute another important risk factor for ART failure. These findings may explain the association of the A6 subtype with the virological failure of LAI CAB/RPV. In our study, approximately 40% of patients had either RPV or CAB DRMs or the HIV A6 subtype with the L74I polymorphism, and up to 5% of patients had both HIV subtype A6 with the L74I polymorphism and RPV or CAB DRMs. In these settings, almost half the patients can be at risk of LAI failure, with at least one risk factor.

The role of the integrase L74I polymorphism in CAB failure remains debated, as the L74I integrase polymorphism has been shown not to differentially impact in vitro sensitivity to CAB across HIV-1 subtype B and A6 integrase genes, and the majority of patients with A6 and L74I maintain virological suppression on the LAI [15,34]. In contrast, the viral failure rate in participants with L74I in subtypes other than A6 does not increase and is similar to that of the overall population (1.8 vs. 1.3%), and this relationship is supported by the absence of participants with HIV-1 subtype B plus L74I and viral failure [15]. Therefore, the effect of L74I on CAB resistance should be considered in the context of A6 subtype characteristics [15,30]. In our study, the L74I polymorphism was present in 86.67% of individuals with subtype A6 HIV infection but only 28.57% of PWH with the B subtype. We did not perform a comparative analysis of LAI CAB/RPV effectiveness in these two cohorts, as this was not the study’s aim. Further research is warranted to understand the interplay of L74I with other subtypes.

#### 4.2.2. DRMs in the A6 HIV-1 Subtype

The prevalence of DRMs in various geographical areas is a complex function of regional ART history, with treatment failure resulting in emergent resistance, transmission networks, and human migration [6,8,12,22,27]. In populations of PWH exposed to ART, especially in high-income countries where the B subtype predominates, the prevalence of transmitted DRMs is greater than in ART-naïve regions and is increasing over time [24,36]. The prevalence of transmitted major HIV-1 DRMs reported worldwide ranges from 5% to 15%; depending on drug class mutations, it is estimated to be 2.0–4.4% and 0.2% for NNRTIs and InSTIs, respectively [23,24,36]. Data concerning southern Russia, where A6 accounted for almost 70% of cases, revealed transmitted NNRTI resistance in 6.4% of cases [26]. Reports state that if treatment failure with CAB/RPV occurs, it is more likely due to RPV with an InSTI mutation rather than an InSTI mutation alone [30,37]. In the epidemiological setting, non-B subtype viruses were found to have more NNRTI DRMs than B subtype viruses, mainly due to the widespread mutation at position E138, which is twice as common among non-B subtype viruses as among B subtype viruses, reaching 8.3–11.9%. Moreover, NNRTI resistance substantially increases the risk of InSTI resistance [24,36,38,39].

Similarly to previous epidemiological reports, in our study, major DRMs were found in 7.56% of all patients (4.48% and 0.56% for NNRTIs and InSTIs, respectively). In accordance with other studies [24,36], subtype B was associated with a greater frequency of all DRMs, including those for InSTI, mainly due to accessory DRMs and polymorphisms (49.06% vs. 32.77% in A6; *p* < 0.05). Interestingly, we detected that the A6 subtype was related to a higher frequency of major mutations, including major DRMs for NNRTI (8.47% vs. 1.89% in the B subtype, *p* < 0.05) and transmitted DRMs (7.34% vs. 1.26% in the B subtype). In studies from Russia and FSU, some acquired DRMs for NNRTI (G190S, K101E, and K103N) were associated with subtype A6 samples, resulting in a significant association of these DRMs in the context of A6 and ART exposure and the possibility of drug-resistant strain transmission [26,40]. Although LAI CAB/RPV is recommended for virologically suppressed patients, the influence of archived DRMs in A6 should also be considered, as the loss time for a DRM can last years, providing the basis for the risk of forming transmitted DRM clusters [23]. Migrants from FSU, owing to HIV-1 subtype A6 transmission with the L74I polymorphism, can be the source of an increased frequency of treatment failure due to drug resistance; however, in this delicate matter bordering origin stigmatization, conclusions should be drawn with great caution. To date, in Poland, war migration has not been proven to enhance the circulation of resistant strains [41].

### 4.3. HIV-1 Subtype A6 in the Context of LAI ART and CAB PrEP

The emergence of drug resistance subtypes, along with a growing number of DRM strains, has led to a growing pool of PWH who cannot be effectively treated with ART [5,15,27]. Moreover, a reduction in the number of drugs used in treatment schemes may facilitate DRM emergence. After approval by the FDA in 2021, the two-drug regimen CAB/PRV is recommended as switch therapy for virologically suppressed patients, and CAB serves as pre-exposure prophylaxis (PrEP) for HIV infection [30,42]. The results from clinical trials support the use of long-acting CAB/RPV in routine clinical practice, as only 1.25% of participants experienced treatment failure with this scheme. Interestingly, the efficacy of CAB/RPV in the treatment of PWH who are unable to achieve viral suppression due to poor adherence has also been confirmed; nevertheless, to date, this ART option, as well as treatment for ART-naïve patients, is still off-label [15,37,42]. On the other hand, more than half of patients with viral failure (53.8%) had subtype A6 with the L74I polymorphism, which necessitated particular precautions in the utilization of LAI in this HIV subtype [15,27]. A recent modeling study suggested that the use of long-acting CAB PrEP in sub-Saharan Africa would be effective; however, it also suggested a possible increase in ART resistance and a reduction in viral suppression rates [43]. To this day, no treatment guidelines have been established concerning these issues.

In our study, approximately 40% of patients had either RPV or CAB DRMs or the HIV A6 subtype with the L74I polymorphism, and up to 5% of patients had both HIV subtype A6 with the L74I polymorphism and RPV or CAB DRMs. In these settings, almost half of the patients can be at risk of LAI failure, with at least one risk factor. Additionally, in our cohort, the A6 subtype was related to a higher frequency of major transmitted DRMs for RPV. The fact that these significant covariates are associated with a potential increased risk of ART failure can be considered at baseline as useful information for clinicians considering long-acting CAB/RPV LAI, as any combination of at least two factors appears to increase failure risk. In conclusion, the emergence and dissemination of the A6 lineage affect the possibility of wide utilization of the first approved LAI CAB/RPV, as they are considered risk factors for the virological failure of this treatment and are common.

These factors may also have a potential negative effect on the efficacy of CAB as PrEP. In our study, the L74I polymorphism was found in 86.67% of the A6 subtypes. For CAB only, our cohort exhibited DRMs in only 1.26–2.82% of PWH, without differences between subtypes. Unfortunately, data regarding the widespread use of LAI CAB/RPV and CAB PrEP in A6 are lacking, and the real-life impact of resistance in regions where A6 is very high remains unknown.

### 4.4. Significance and Limitations of the Study

The data obtained in our study are epidemiologically relevant because they originate from patients entering standard clinical care and were not included in pharmaceutical trials. We present the epidemiological and genetic characteristics of the increasing prevalence of HIV subtype A6 in the context of newly implemented LAI ART. Another advantage of this study is the incorporation of InSTI genotyping in the A6 subtype, which was not available in most previous trials. Thus, this study provides real-life data on population representatives in areas with knowledge gaps.

Now, given the recent implementation of CAB/RPV LAI schemes, a discussion arises as to whether the L74I polymorphism and A6 subtype may influence treatment recommendations, as they both constitute risk factors for ART failure with CAB and RPV. To date, no recommendations have been made concerning this issue, but there is growing evidence that this topic may affect future recommendations, in which case this study can provide one of the arguments.

The analysis of sequencing data always results in knowledge gaps regarding the associations of new mutations with drug resistance or the occurrence of relevant mutations outside regions targeted by routine resistance assays. This risk is especially high when new drugs are introduced to the schemes, such as a new generation of InSTI in long-acting formulations. Moreover, the study did not examine the genetic context of the A6 subtype in connection with ART effectiveness via prospective patient observations. An experimental trial could provide more accurate insights into CAB and RPV effectiveness in this high-risk population.

## 5. Conclusions

Long-lasting selective pressure by ART and HIV genetic diversity demands constant epidemiological surveillance, even though new drugs have high genetic barriers. This is especially important for regimens with a reduced number of drugs and in the setting of HIV epidemics caused by strains with intrinsic resistance, such as LAI ART and HIV-1 subtype A6, respectively. The increasing number of A6 infections in Poland is evidence of the epidemic spread of the A6 subtype outside the FSU region. Owing to the frequent presence of the L74I polymorphism and DRMs in the A6 subtype, almost half of PWH in Poland can be at risk of LAI CAB/RPV failure. Continued subtyping and screening for DRMs are crucial for LAI prophylaxis and treatment.

## Figures and Tables

**Table 1:** Baseline study group characteristics.

	All Patients (n = 357)	A6 Subtype (n = 177)	B Subtype (n = 159)	*p*
Total number (n (%))	357 (100.00)	177 (49.58)	159 (44.54)	0.177
Age (mean (SD))	34.31 (10.61)	34.15 (10.75)	34.44 (10.94)	0.528
Male sex (n (%))	298 (83.47)	138 (77.97)	141 (88.68)	0.008
Female sex (n (%))	59 (16.53)	39 (22.03)	18 (11.32)	0.009
FSU nationality (n (%))	102 (28.57)	79 (44.63)	23 (14.47)	<0.001
Polish nationality (n (%))	247 (69.11)	96 (54.24)	131 (82.39)	<0.001
MSM (n (%))	238 (66.67)	104 (58.76)	118 (74.21)	0.003
Heterosexual (n (%))	86 (24.09)	58 (32.77)	24 (15.09)	<0.001
IDU (n (%))	22 (6.16)	10 (5.65)	12 (7.55)	0.493
Vertical (n (%))	11 (3.08)	5 (2.82)	5 (3.14)	0.863
Recent HIV infection (n (%))	70 (19.61)	34 (19.21)	31 (19.50)	0.947
AIDS (n (%))	61 (17.09)	30 (16.95)	30 (18.87)	0.647

**Table 2:** The presence of major and accessory HIV drug resistance mutations in the analyzed population.

	All Patients (n = 357)	A6 Subtype (n = 177)	B Subtype (n = 159)	*p*
All mutations				
Any mutation (n (%))	143 (40.06)	58 (32.77)	78 (49.06)	0.002
Any major mutation (n (%))	36 (10.08)	24 (13.56)	11 (6.92)	0.047
Any accessory mutation (n (%))	118 (33.05)	42 (23.73)	72 (45.28)	<0.001
InSTI mutations				
Any InSTI mutation (n (%))	44 (12.32)	9 (5.08)	32 (20.13)	<0.001
Any major InSTI mutation (n (%))	4 (1.12)	2 (1.13)	2 (1.26)	0.914
Any accessory InSTI mutation (n (%))	40 (11.20)	7 (3.95)	30 (18.87)	<0.001
Any InSTI polymorphism (n (%))	12 (3.36)	0 (0.00)	9 (5.66)	0.006
NNRTI mutations				
Any NNRTI mutation (n (%))	58 (16.25)	29 (16.38)	27 (16.98)	0.883
Any major NNRTI mutation (n (%))	19 (5.32)	15 (8.47)	3 (1.89)	0.007
Any accessory NNRTI mutation (n (%))	39 (10.92)	14 (7.91)	24 (15.09)	0.038
Any NNRTI polymorphism (n (%))	25 (7.00)	13 (7.34)	5 (3.14)	0.088
HIV drug resistance				
Transmitted, major all (n (%))	27 (7.56)	18 (10.17)	9 (5.66)	0.129
Transmitted major to INSTI (n (%))	2 (0.56)	1 (0.56)	1 (0.63)	0.939
Transmitted major to NNRTI (n (%))	16 (4.48)	13 (7.34)	2 (1.26)	0.007
Acquired, major all (n (%))	9 (2.52)	6 (3.39)	3 (1.89)	0.269
Acquired major to INSTI (n (%))	2 (0.56)	1 (0.56)	1 (0.63)	0.939
Acquired major to NNRTI (n (%))	3 (0.84)	2 (1.13)	1 (0.63)	0.626

**Table 3:** The presence of HIV drug resistance mutations to cabotegravir and rilpivirine in the analyzed population.

	All Patients (n = 357)	A6 Subtype (n = 177)	B Subtype (n = 159)	*p*
Cabotegravir mutations				
All cabotegravir mutations (n (%))	10 (2.80)	5 (2.82)	4 (2.52)	0.861
Major cabotegravir mutations (n (%))	5 (1.40)	3 (1.69)	2 (1.26)	0.741
Accessory cabotegravir mutations (n (%))	5 (1.40)	2 (1.13)	2 (1.26)	0.914
Cabotegravir polymorphisms (n (%))	12 (3.36)	0 (0.00)	9 (5.66)	0.006
Rilpivirine mutations				
All rilpivirine mutations (n (%))	45 (12.61)	19 (10.73)	24 (15.09)	0.232
Major rilpivirine mutations (n (%))	11 (3.08)	9 (5.08)	1 (0.63)	0.016
Accessory rilpivirine mutations (n (%))	34 (9.52)	10 (5.65)	23 (14.47)	0.007
Rilpivirine polymorphisms (n (%))	25 (7.00)	13 (7.34)	5 (3.14)	0.088

## Data Availability

Data supporting reported results can be found in the Department of Infectious Diseases, Tropical Diseases and Hepatology, Medical University of Warsaw, Poland.
